# A pivotal study on the novel cutting balloon KCB01 in patients with coronary artery disease

**DOI:** 10.1007/s12928-025-01205-7

**Published:** 2025-10-17

**Authors:** Yoshisato Shibata, Yoshiaki Ito, Shigeru Nakamura, Koichi Kishi, Yuji Oikawa, Takashi Muramatsu, Gaku Nakazawa, Hisashi Koga, Kenichi Sakakura, Takuya Shida, Masato Nakamura

**Affiliations:** 1 Department of Cardiology, Miyazaki Medical Association Hospital, Miyazaki, Japan; 2 Department of Cardiology, Saiseikai Yokohama City Eastern Hospital, Yokohama, Japan; 3 Cardiovascular Center, Kyoto Katsura Hospital, Kyoto, Japan; 4 Department of Cardiology, Tokushima Red Cross Hospital, Tokushima, Japan; 5 Department of Cardiovascular Medicine, The Cardiovascular Institute, Tokyo, Japan; 6 Department of Cardiology, Fujita Health University Hospital, Aichi, Japan; 7 Department of Cardiology, Kindai University Hospital, Osaka, Japan; 8 Department of Cardiology, Cardiovascular Center, Shin-Koga Hospital, Fukuoka, Japan; 9 Division of Cardiovascular Medicine, Saitama Medical Center, Jichi Medical University, Saitama, Japan; 10 Medical Solutions Vehicle, Kaneka Corporation, Osaka, Japan; 11 Division of Minimally Invasive Treatment in Cardiovascular Medicine, Toho University Ohashi Medical Center, 2-22-36, Ohashi, Meguro-Ku, Tokyo, 153-8515 Japan

**Keywords:** Clinical trial, Percutaneous coronary intervention, Cutting balloon, Vessel preparation

## Abstract

The KCB01 (KIZASHI^™^) is a novel cutting balloon catheter with blades integrally formed with the balloon. It is designed to overcome the drawbacks of conventional cutting balloons in terms of lesion delivery, crossability and procedural complications while maintaining the same dilation capacity. To evaluate the efficacy and safety of the KCB01, a prospective, open-label, single-arm study was conducted at nine sites in Japan on percutaneous coronary intervention-eligible patients with stenotic lesions that exhibited residual indentation even after conventional balloon dilation at nominal pressure. Seventy-six patients were enrolled between November 2022 and June 2023, and 73 eligible patients were included in the primary analysis. The mean age was 73.5 ± 7.4 years, and 79.5% of the patients were male. The target lesion characteristics included bifurcation lesions (42.5%), in-stent restenosis lesions (20.5%), and severely calcified lesions (54.8%). The primary endpoint was procedural success, defined as the KCB01 crossing the target lesion, no residual indentation, and < 50% residual stenosis rate after KCB01 dilation (assessed using quantitative coronary angiography). The angiographic images obtained during the procedure were evaluated in a core laboratory. The procedural success rate was 87.7% (95% confidence interval [CI]: 77.9–94.2), with the lower limit of the 95% CI (77.9%) exceeding the performance goal of 76.5%. Clinical success rate was achieved in 94.5% of cases (95% CI: 86.6–98.5%), with no major adverse cardiac events reported until discharge. Furthermore, no device deficiencies related to the KCB01 were observed. These results suggest the safety and efficacy of the KCB01.

**Graphical abstract:**

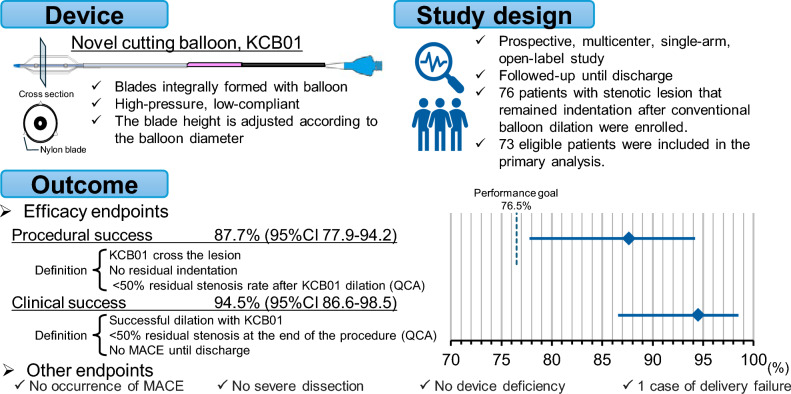

**Supplementary Information:**

The online version contains supplementary material available at 10.1007/s12928-025-01205-7.

## Introduction

With the widespread use of drug-eluting stents (DESs) and drug-coated balloons (DCBs), the importance of vessel preparation in improving outcomes has been widely recognized [1, 2]. Devices for vessel preparation include cutting balloons, scoring balloons, rotational atherectomy, orbital atherectomy, and intravascular lithotripsy, each of which is used for different lesion types based on their characteristics [3–7]. Cutting balloons are used to expand vascular stenosis, which are difficult to treat using conventional balloons, while inhibiting serious dissection. The principle of action of cutting balloons is that when the balloon is pressurized and expanded, the blades folded into the balloon emerge and are pressed against the plaque to make controlled dissections in the coronary artery, achieving dilation [8]. Cutting balloons equipped with metal blades can address the following issues [3, 8]: (1) reduced flexibility of the balloon leading to poor lesion delivery, (2) a larger balloon profile resulting in poor crossability, (3) risk of blade detachment, and (4) risk of vessel perforation. To address these issues, a novel cutting balloon, the KCB01 (KIZASHI^™^, Kaneka Corporation, Osaka, Japan), with integrated resin blades and a balloon structure was developed. The KCB01 overcomes the drawbacks of conventional cutting balloons in terms of lesion delivery and crossability, and its design prevents dissection at the balloon edges because it is less prone to dog-bone effects. This study aimed to evaluate the performance of these features.

## Methods

### Study design

This was a prospective, open-label, single-arm trial. The patients were enrolled from nine sites in Japan. This study was conducted in compliance with the ethical principles of the Declaration of Helsinki and Good Clinical Practice guidelines for medical devices. The study was registered with the Japan Registry of Clinical Trials (jRCT) under the registration number jRCT2032220438 and was conducted with the approval of the institutional review board at each participating site.

### Study device

**Fig. 1:**
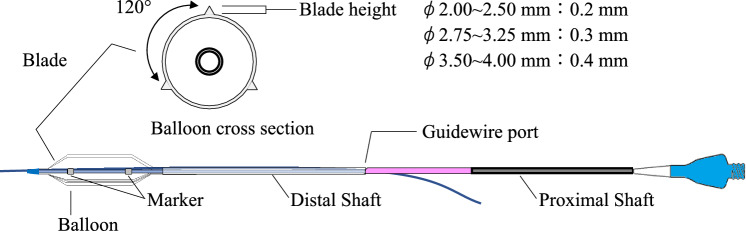
Diagram of the KCB01

The KCB01 (KIZASHI^™^) is a cutting-type balloon catheter with nylon blades integrally formed with the balloon. The nominal pressure is 20 atm and the rated burst pressure (RBP) is 24 atm. The balloon is designed to be low-compliance, meaning that its outer diameter does not significantly increase, even under high pressure. The blades are arranged circumferentially at 120-degree intervals, and the blade height is adjusted according to the balloon diameter. The compatible guiding catheter is 5 Fr for balloon diameters of 3.0 mm or less, and 6 Fr for balloon diameters of 3.25 mm or more. The compatible guidewire size is 0.014 inches (Fig. 1).

### Study population

The main inclusion criteria were as follows: (1) patients with stenotic lesions with > 90% stenosis, or lesions that are considered the cause of stable exertional angina classified as Class II or higher according to the Canadian Cardiovascular Society classification, or lesions that have been confirmed through functional testing to be the cause of functional ischemia; (2) patients with a target lesion located in a vessel with a diameter between 2.0 and 4.0 mm; (3) patients with a target lesion length of 38 mm or less; and (4) patients with a target lesion with residual indentation by conventional balloon nominal pressure dilation. The main exclusion criteria were as follows: (1) patients with a left ventricular ejection fraction of less than 40% within 30 days prior to the procedure; (2) patients with New York Heart Association Class III or IV heart failure; (3) patients with a target lesion in the unprotected left main coronary artery; 4) patients whose target lesion is in a vessel that can only be reached through the saphenous vein or arterial bypass graft; (5) patients with a target lesion located at or distal to a vessel bend greater than 45 degrees; (6) patients with excessive tortuosity in the target vessel; (7) patients with thrombus or ulceration in the target vessel; and (8) patients with angiographic evidence of severe dissection in the target vessel after conventional balloon dilation. All inclusion and exclusion criteria are listed in the Supplemental Table S1.

Information. Only one target lesion was treated with the KCB01; if multiple lesions met the criteria, the physician selected the target lesion. Patients who met the inclusion criteria and did not violate the exclusion criteria were considered eligible, and enrollment occurred when the KCB01 was inserted into the guiding catheter. Patients judged by the independent data monitoring committee (IDMC) to have no residual indentation by conventional balloon nominal pressure dilation after core lab analysis were excluded from the efficacy analysis.

### Procedure

Access sites (transradial approach or transfemoral approach) were not specified and left to the physician’s discretion. The selection of the KCB01 size was also at the discretion of the physician, and the use of a guide extension catheter was not restricted. If crossing the lesion with the KCB01 was difficult, pre-dilation with the conventional balloon used for the indentation check or a conventional balloon of the same or smaller size than the KCB01 was allowed. The dilation pressure of the KCB01 was set within the RBP range at the physician’s discretion, and there was no limit on the number of dilations.

No other device treatment was allowed for revascularization of the target lesion between patient enrollment and post-KCB01 angiography. After dilation with the KCB01, the use of any device or therapy other than conventional balloons, DCBs, or stents was prohibited. If pre-dilation with the KCB01 was insufficient, the residual stenosis was severe, and the physician judged that completing the procedure was difficult, the use of a device such as a rotational atherectomy or orbital atherectomy system was permitted, but the procedure was judged as unsuccessful.

There were no specific regulations regarding the selection of DES or DCB after KCB01 dilation, and the physician selected the device according to the extent of the dissection. Patient follow-up was carried out until the time of discharge.

### Angiographic analysis

**Fig. 2:**
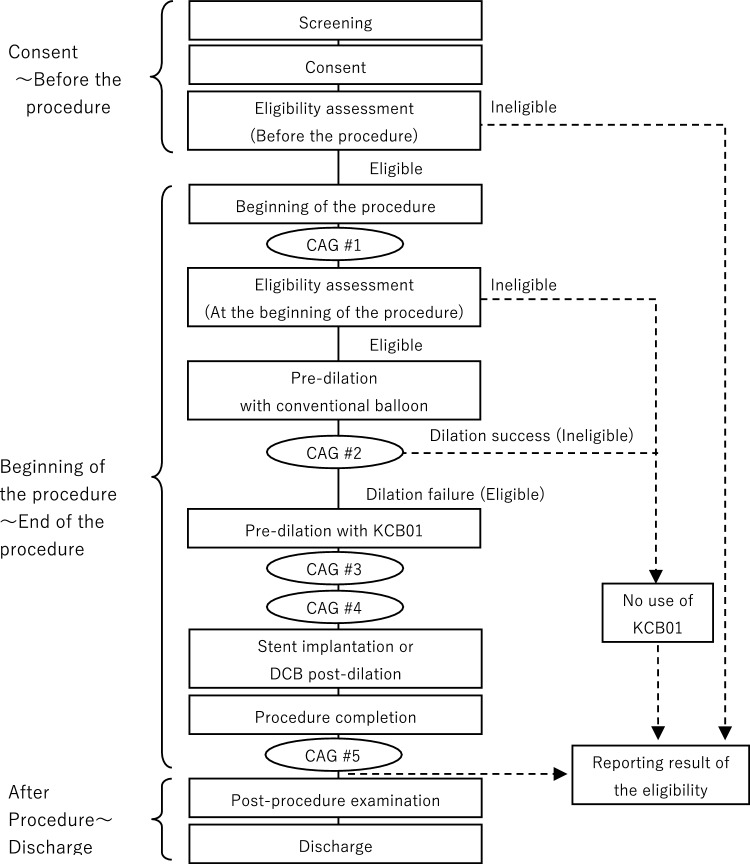
Procedure flow chart, detailing the steps from consent to discharge, including eligibility assessment, pre-dilation, stent implantation, and post-procedure examination. CAG #1 pre-procedure; CAG #2 at the time of indentation check with a conventional balloon; CAG #3 at the time of indentation check with the KCB01; CAG #4 after KCB01 dilation; CAG #5 upon completion of the procedure.*CAG* coronary angiography; *DCB* drug-coated balloon

Angiographic analysis was performed at pre-procedure, during conventional balloon dilation for eligibility assessment (at the time of indentation check with a conventional balloon), during KCB01 dilation (at the time of indentation check with the KCB01), after KCB01 dilation, and after the procedure (Fig. 2).

**Fig. 3:**
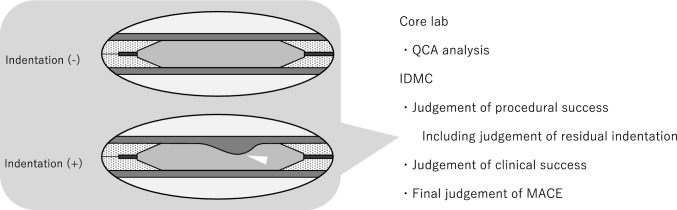
Core lab and independent data monitoring committee (IDMC) indentation judgement, showing the process of quantitative coronary angiography (QCA) analysis, procedural success judgement, clinical success judgement, and final judgement of major adverse cardiac event (MACE)

All angiographic images were analyzed by the core laboratory (CARDIOCORE JAPAN, Tokyo, Japan) for qualitative lesion and quantitative coronary angiography (QCA) analyses (Supplemental Table S2). The main evaluations at the core lab included target lesion site, lesion morphology (e.g., American Heart Association [AHA] lesion classification, in-stent restenosis [ISR] lesion, ostial lesion, bifurcation lesion, calcification, and presence of dissection), dissection after KCB01 dilation, in-segment minimum lumen diameter, and in-segment residual stenosis rate. Calcification was classified as none/mild (not visible on CAG), moderate (radiopacities noted only during the cardiac cycle before contrast injection), and severe (radiopacities noted without cardiac motion before contrast injection generally compromising both sides of the arterial lumen) [9]. The evaluation results from the core lab, including judgments on the presence or disappearance of indentations impacting eligibility and procedural success, were reviewed by the IDMC for final determination. The evaluations at the core lab and IDMC are shown in Fig. 3.

### Endpoint

The primary endpoint was the procedural success rate, defined as the KCB01 crossing the target lesion, no residual indentation, and a < 50% residual stenosis rate after KCB01 dilation (assessed by QCA). Secondary endpoints included the clinical success rate, major adverse cardiac event (MACE) incidence, in-segment minimum lumen diameter, in-segment residual stenosis rate, grade of dissection based on National Heart, Lung, and Blood Institute (NHLBI) classification, incidence of cardiac death, incidence of myocardial infarction, and target vessel revascularization (TVR) incidence. Clinical success was defined as successful dilation with the KCB01, residual stenosis less than 50% at the end of the procedure (evaluated by QCA), and the absence of MACE until discharge. MACE included cardiac death, nonfatal myocardial infarction, and revascularization based on clinical findings. The definitions of the other endpoints are provided in the Supplemental Table S3.

The final determinations of procedural success, clinical success, NHLBI classification, and MACE were performed using the IDMC.

Safety endpoints included the nature and incidence of adverse events and device deficiencies, laboratory data, vital signs, and 12-lead electrocardiograms (ECGs).

### Statistical analysis

Continuous variables were presented as mean ± standard deviation, and binary data for efficacy evaluation were expressed as percentages and 95% confidence intervals (CIs; Clopper-Pearson).

The performance goal was set at 76.5% based on the procedural completion rate for cutting balloons from a previous domestic clinical study [10] and the percutaneous coronary intervention (PCI) success rate reported before the standardization of stent treatment [11]. Differences in the target lesions (such as the proportion of calcified lesions) between those reports and the target lesions expected in this study were considered.

For the primary endpoint of the procedural success rate, the efficacy of the KCB01 was confirmed if the lower limit of the two-sided 95% CI by Clopper-Pearson exceeded the set performance goal. Assuming an expected procedural success rate of 90% for the KCB01, a one-sided significance level of 2.5%, and a power of 80%, the required sample size was determined to be 63. Considering a dropout margin of approximately 10%, the target number of patients was set to 70.

The primary analysis set for efficacy evaluation was the full analysis set (FAS). Cases deemed not to meet the inclusion criteria of the IDMC, with no efficacy evaluation data obtained, were excluded from the FAS. The data management and statistical analysis were conducted by the contract research organization (RPM Co., Ltd., Tokyo, Japan). The software used for the statistical analysis included SAS Release 9.4 TS1M4 (× 64 version) and Microsoft Excel for Microsoft 365 (version 2301). The aggregation of adverse events and device deficiencies included all cases in which the KCB01 was inserted into the guiding catheter.

## Results

### Clinical characteristics

**Fig. 4:**
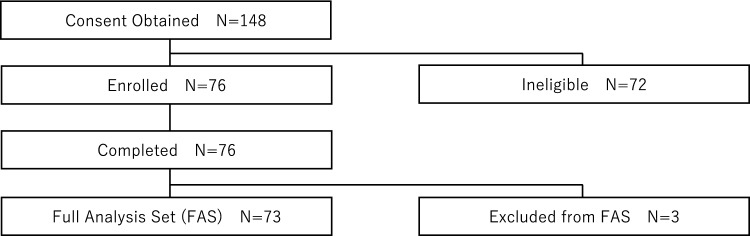
Patient flow chart

Consent was obtained from 148 patients between November 2022 and June 2023. Each patient was confirmed to meet the inclusion criteria and not violate the exclusion criteria by the physicians, resulting in 76 cases being enrolled and 73 cases being included in the FAS (Fig. 4). Table 1 shows the baseline demographic and other baseline characteristics. The mean age was 73.5 ± 7.4 years, with 79.5% being male, 84.9% having hypertension, 87.7% having dyslipidemia, 31.5% having diabetes mellitus, 56.2% having a history of PCI, and 6.8% being dialysis patients.

**Table 1:** Demographic and other reference value characteristics at baseline

Patient background N = 73 (%)	
Age, years	73.5 ± 7.4
Male	58 (79.5)
Hypertension	62 (84.9)
Dyslipidemia	23 (31.5)
Diabetes mellitus	23 (31.5)
Past smoker	47 (64.4)
Current smoker	5 (6.8)
Prior PCI	41 (56.2)
Prior coronary artery bypass grafting	1 (1.4)
Dialysis	5 (6.8)
CCS classification	
0	1 (1.4)
I	8 (11.0)
II	57 (78.1)
III	8 (6.2)
IV	1 (1.4)
LVEF	61.7 ± 9.5
NYHA classification	
I	48 (65.8)
II	24 (32.9)
III	0 (0.0)
IV	0 (0.0)

*PCI* percutaneous coronary intervention; *CCS* Canadian Cardiovascular Society; *LVEF* left ventricular ejection fraction; NYHA New York Heart Association.

### Angiographic and procedural characteristics

Lesion information from before the procedure is presented in Table 2. The ostial lesions, bifurcation lesions, and severe calcifications were 5.5, 42.5, and 54.8%, respectively. The ISR rate was 20.5%.

**Table 2:** Procedure information: Pre-procedre

Procedure information: Pre-procedure N = 73 (%)	
Ostial lesion	4 (5.5)
Bifurcation lesion	31 (42.5)
Calcification	
None/mild	12 (16.4)
Moderate	21 (28.8)
Severe	40 (54.8)
Bend of lesion	
< 45°	72 (98.6)
45° ~ 90°	1 (1.4)
90°≦	0 (0.0)
Tortuosity	
None	73 (98.6)
Moderate	1 (1.4)
Severe	0 (0.0)
Eccentric lesion	22 (30.1)
ISR	15 (20.5)
AHA lesion classification	
A	1 (1.4)
B1	13 (17.8)
B2	43 (58.9)
C	16 (21.9)
Reference Diameter(mm)	2.893 ± 0.432
In-segment minimum lumen diameter (mm)	0.904 ± 0.383
In-segment residual stenosis (%)	63.9 ± 13.0
Lesion length	
< 10 mm	3 (4.1)
10 mm ~ 20 mm	45 (61.6)
20 mm <	25 (34.2)
Access sites	
Radial approach	65 (89.0)
Femoral approach	8 (11.0)
Guiding catheter size	
6 Fr	50 (68.5)
7 Fr	23 (31.5)

*ISR* in-stent restenosis; *AHA* American Heart Association.

Table 3 shows the information at the time of the indentation check with a conventional balloon, the indentation check with the KCB01, and after KCB01 dilation. The mean pressure applied during the indentation check using the conventional balloon was 6.3 ± 1.3 atm. The mean maximum pressure during the indentation test with the KCB01 was 22.3 ± 2.0 atm. The diameters of the KCB01 used for dilation and the conventional balloon used for the indentation check were identical in all cases.

**Table 3:** Procedure information: Dilation pressure at each time point, indentation, grade of dissection

Procedure information: Dilation pressure at each time point, indentation, grade of dissection N = 73 (%)		
	At the time of conventional balloon dilation	At the time of KCB01 dilation
Pressure (actual maximum pressure applied) (atm)	6.4 ± 1.3	22.3 ± 2.0
Indentation		
-	0 (0.0)	67 (91.8)
+	73 (100.0)	5 (6.8)
NA^※^	–	1 (1.4)
※Case that KCB01 did not cross		
Grade of dissection based on NHLBI classification		
Type A	2 (2.7)	23 (31.5)
Type B	2 (2.7)	19 (26.0)
Type C	0 (0.0)	1 (1.4)
Type D	0 (0.0)	0 (0.0)
Type E	0 (0.0)	0 (0.0)
Type F	0 (0.0)	0 (0.0)

*NA* not available; *NHLBI* National Heart, Lung, and Blood Institute.

It was found that 58.9% of cases had dissections after KCB01 dilation, with 38.4% classified as NHLBI Type A, 26.0% as Type B, 1.4% as Type C, and none as Type D or higher.

Post-procedure information is presented in Table 4. The use of stents was 61.6%, the use of DCB was 31.5%, and the use of both stents and DCB was 1.4%.

**Table 4:** Procedure information: At completion of the procedure

Procedure information: At completion of the procedure N = 73 (%)	
Use of stent or DCB^※^	
No	3 (4.1)
Yes	69 (94.5)
Stent	45 (61.6)
DCB	23 (31.5)
Stent and DCB	1 (1.4)
Post-dilation after stent deployment^※^	27 (37.0)
※Cases where the KCB01 did not cross are excluded from aggregation	

*DCB* drug-coated balloon.

### Efficacy endpoints

The efficacy endpoints are listed in Table 5. The number of procedural successes was 64, with a procedural success rate of 87.7 and a 95% CI of 77.9–94.2%. Unsuccessful cases included one case of KCB01 delivery failure, five cases of residual indentation at the time of KCB01 dilation, and three cases of failure to achieve less than 50% residual stenosis after KCB01 dilation. Among the residual indentation cases, two involved the use of rotational atherectomy as a bailout. The details of angiographic characteristics of the procedural failure cases were depicted in Supplemental Table S4. The number of clinical successes was 69, with a clinical success rate of 94.5 and a 95% CI of 86.6–98.5%. Unsuccessful cases included one case of KCB01 delivery failure, two cases of unsuccessful dilation with the KCB01 (both of which required rotational atherectomy as a bailout), and one case of failure to achieve less than 50% residual stenosis at the completion of the procedure. No occurrences of MACE, cardiac death, myocardial infarction, or TVR were observed until discharge.

**Table 5:** Efficacy endpoints

Efficacy endpoints N = 73 (%)	
Procedural success (%)	64 (87.67)
95% confidence interval	77.88–94.20
Clinical success (%)	69 (94.52)
95% confidence interval	86.56–98.49
QCA analysis	
Pre-procedure N = 73	
Reference diameter (mm)	2.489 ± 0.479
In-segment minimum lumen diameter (mm)	0.904 ± 0.383
In-segment residual stenosis rate (%)	63.9 ± 13.0
At the time of indentation check with a conventional balloon N = 73	
Reference diameter (mm)	2.388 ± 0.408
In-segment minimum lumen diameter (mm)	1.594 ± 0.446
In-segment residual stenosis rate (%)	33.6 ± 12.8
At the time of indentation check with the KCB01 N = 73	
Reference diameter (mm)	2.493 ± 0.394
In-segment minimum lumen diameter (mm)	2.245 ± 0.404
In-segment residual stenosis rate (%)	10.1 ± 5.1
After KCB01 dilation N = 71	
Reference diameter (mm)	2.328 ± 0.393
In-segment minimum lumen diameter (mm)	1.657 ± 0.367
In-segment residual stenosis rate (%)	28.7 ± 12.1
At the completion of the procedure N = 73	
Reference diameter (mm)	2.680 ± 0.550
In-segment minimum lumen diameter (mm)	2.165 ± 0.526
In-segment residual stenosis rate (%)	19.3 ± 9.7
Distribution of residual stenosis rate	
At completion of the procedure N = 73	
≦30%	65 (89.0)
30% ~ 50%	7 (9.6)
50%≦	1 (1.4)

*QCA* quantitative coronary angiography.

### Safety endpoints

In this study, 10 patients (13.2%) experienced 13 adverse events. Among these patients, one case each of coronary artery dissection and coronary artery perforation was reported; however, both were attributed to other devices, and no association with KCB01 was identified. All other cases were nonserious and there were no adverse events with a relationship with the KCB01 (0.0%). No device deficiencies were observed in the KCB01. Apart from those judged as adverse events, there were no significant changes in the clinical laboratory data, vital signs, or 12-lead ECGs. A list of adverse events is provided in Supplemental Table S5.

## Discussion

The KCB01 was developed with a design that improves trackability while maintaining the same dilation capacity as that of an already approved cutting balloon. The main differences are as follows: (1) the blades are made of nylon and formed integrally with the balloon, (2) the blade height varies by size and is higher than that of the approved models, and (3) the balloon is low-compliant and has a high pressure resistance. The procedural success rate, which was the primary endpoint of this study, was 87.7% (64/73 cases, 95% CI: 77.9–94.2%). As the lower limit of the 95% CI (77.9%) exceeded the performance goal of 76.5%, the non-inferiority of the KCB01 to the performance goal was verified.

In this study, 10 patients (incidence rate: 13.2%) experienced adverse events. Among them, there were no adverse events (incidence rate of 0.0%) with a relationship with the KCB01; they were estimated to be related to other devices or procedures, or to have occurred accidentally. In addition, no device deficiencies were observed for the KCB01 in this study.

### Dilation capacity

As for the secondary endpoints, the in-segment minimum lumen diameter and in-segment residual stenosis rate, a previous study [10] reported the results of balloon angioplasty with a cutting balloon in 72 cases where the procedure was completed solely with a cutting balloon for small-vessel diseases. According to the reported study, the post-procedural in-segment minimum lumen diameter and in-segment residual stenosis rate were 1.80 ± 0.32 mm and 28.6 ± 12.3%, respectively. In this study, the in-segment minimum lumen diameter and in-segment residual stenosis rate after KCB01 dilation were 1.66 ± 0.37 mm and 28.7 ± 12.1%, respectively.

Comparing the reported study with this study, the in-segment residual stenosis rate was almost the same in both studies. However, while the reported study excluded calcified lesions, 83.6% (61 patients) of the patients in this study had moderately or severely calcified lesions. Therefore, the subjects in this study had more challenging lesions to dilate than those in the previous study. In contrast, the dilation pressures were significantly higher for the KCB01. Therefore, although a direct comparison is not possible, the similar in-segment residual stenosis rate suggests that the performance of the KCB01 is comparable to that of existing cutting balloons.

In this study, procedural failures with the KCB01 were attributed to residual indentation at the time of KCB01 dilation in five cases and failure to achieve less than 50% residual stenosis after KCB01 dilation in three cases. These lesions included seven cases of severe calcification and one case of moderate calcification. In two cases where residual indentation persisted after KCB01 dilation, a rotational atherectomy device was used prior to the use of DES or DCB. In other cases, the physician judged that the indentation had not been observed and proceeded with the procedure using DES or DCB, although core lab evaluation later confirmed residual indentation in these cases. Similar findings were reported in a Rota-Shock study [12], where a certain frequency of bailout use in rotational atherectomy was noted.

In this study, the use of other vessel preparation devices was restricted to evaluate the performance of the KCB01 as accurately as possible. However, in clinical practice, combinations of other pre-treatment devices and cutting balloons may be considered. The outcomes of this combined use remain an issue that should be clarified in future studies.

### Dissection

In the present study, the number of cases found with dissection increased following KCB01 dilation (4 cases at the time of conventional balloon dilation and 53 cases after KCB01 dilation). However, the most severe type of dissection was Type C (1.4%, n = 1). KCB01 did not cause large dissection or extension of longitudinal dissection. This may be due to the balloon being highly low compliant. Umeda et al. [10] reported that major dissection (equivalent to NHLBI classification Type D or higher) occurred in 6.8% (6/88) of cases. Another report also indicated that Type D, E, and F dissections occurred in 2.1% (5/229) of patients with ISR lesions [13]. In this study, 59% of the lesions were classified as AHA Type B2/C, whereas 80.8% were classified as AHA Type B2/C. Additionally, the maximal inflation pressure of cutting balloons in the study was 9.9 ± 2.6 atm, which was lower than that of the KCB01 in this study. The reported studies using cutting balloons showed an expansion pressure 3–4 atm higher than the nominal pressure (6 atm), but still lower than that for the KCB01. Cutting balloons are equipped with blades on compliant balloons, and even with a slight increase in pressure, the dog-bone phenomenon is likely to occur in areas without blades, potentially causing dissection at the balloon edges. High-pressure dilation is thought to cause significant vessel damage and larger dissections. However, in this study, the median inflation pressure of the KCB01 was 24 atm, and high-pressure dilation reaching the RBP was performed in most cases; however, the incidence of major dissections was low. One reason for this could be that the KCB01, being low-compliant, is less likely to cause the dog-bone phenomenon, even at high pressures, leading to fewer large dissections. Considering these results, the incidence of dissection during KCB01 dilation was not expected to be higher.

### Crossability

In this study, there was one case of delivery failure. The lesion in this case was a de novo total occlusion lesion, where the initial attempt to cross a φ2.00 × 15 mm KCB01 was unsuccessful. Subsequently, a φ2.00 × 10 mm KCB01 was used, and it successfully crossed and dilated the lesion (this case was counted as a procedural failure). The rate of KCB01 delivery failure was 1.4%. In a study that compared the delivery success rate of cutting and scoring balloons, focusing on delivery success as the primary endpoint, the delivery success rate of cutting balloons (defined as successful passage through the target lesion immediately after intravascular ultrasound or optical coherence tomography) was 90.8% [14]. This study specifically enrolled patients with calcified lesions, and rotational atherectomy was performed in 18% of cases. Lesions classified as AHA Type B2/C accounted for 86% of the cases. Previous reports on delivery failure rates include Umeda et al. with 5.9% (5/88 cases) [10], Albiero et al. with 5.2% (12/229 cases) [13], Ozaki et al. with 1.5% (4/260 cases) [15], and a recent report by Mangieri et al. with 2.3% (1/44 cases) [16]. In this study, 83.6% of the treated lesions exhibited moderate-to-severe calcification, and 80.8% of the lesions were classified as AHA Type B2/C. The variability in delivery failure rates reported in these studies could be explained by differences in the era of the study, lesion selection, and types of devices allowed for use. Nonetheless, the 1.4% crossability failure rate for the KCB01 in this study is comparable to those in existing reports.

### Limitations

In local clinical practice, severely calcified lesions are often treated with debulking devices, such as rotational or orbital atherectomy before using cutting balloons. However, this study aimed to evaluate the performance of the KCB01; therefore, the use of debulking devices before the KCB01 was prohibited. This implies that the sequence used in this study differs from that used in actual clinical practice. Nonetheless, studies comparing rotational atherectomy, high-pressure dilation, and cutting balloons for calcified lesions have reported no significant differences in device performance, indicating that the superiority of lesion preparation devices for calcified lesions remains unclear [17]. This study was designed and conducted as a single-arm study, not a randomized controlled trial, and was relatively small in scale. Therefore, there are limitations when comparing the KCB01 with other vessel preparation devices, such as high-pressure dilation with non-compliant balloons, other cutting balloons, or scoring balloons. Moreover, this study was conducted exclusively in a Japanese population, and evaluation in other populations is necessary. Finally, intracoronary imaging assessment was not reported in the present study.

## Conclusion

Based on this study, the effectiveness and safety of the new cutting balloon KCB01 for coronary artery stenotic lesions that are difficult to dilate with a conventional balloon using nominal pressure were verified. Further studies are required to compare its use in conjunction with other devices.

## Supplementary Information

Below is the link to the electronic supplementary material.

Supplementary file (DOCX 38 KB)

## Data Availability

Data from this study will not be made available to others because of ownership by the sposor.
