# Supplementary material for: A pivotal study on the novel cutting balloon KCB01 in patients with coronary artery disease
Source: Cardiovasc Interv Ther. 2025 Oct 17;41(1):89–99. doi: 10.1007/s12928-025-01205-7 (PMC12783245; doi:10.1007/s12928-025-01205-7)
Supplement: Supplementary file 1 — Supplementary file (DOCX 38 KB) [file 12928_2025_1205_MOESM1_ESM.docx]

# Supplemental information

## Table 1: All inclusion and exclusion criteria

## Table: Angiographic analysis core lab evaluation items

## Table 3. Definitions

## Table 4 Lesion characteristics of the procedural failure cases

## Table 5. List of all adverse events

## Table 1: All inclusion and exclusion criteria

| Inclusion criteria |
| --- |
| Japanese patients aged 20 years or older |
| Percutaneous coronary intervention (PCI)-eligible patients with lesions that physicians consider difficult to dilate with a conventional balloon |
| Patients with any of the following lesions  Stenotic lesions with >90% stenosis  Stenotic lesions thought to be the cause of stable exertional angina (patients with a confirmed diagnosis of Class II or higher in the Canadian Cardiovascular Society classification)  Stenotic lesion that has been tested for functional ischemia and confirmed as the cause of functional ischemia |
| Patients whose target lesion has a vessel diameter between 2.0 mm and 4.0 mm |
| Patients whose target lesion has a length of 38 mm or less |
| Patients whose target lesion has a diameter stenosis greater than 75% |
| Patients with a 0.014-inch guidewire passing through the target lesion |
| Patients whose target lesion has residual indentation by conventional balloon nominal pressure dilation |
| Patients who have been fully informed of the purpose and content of the KCB01 and the study, voluntarily volunteer, and provide written consent to participate in the study |
|  |
| Exclusion criteria |
| Patient with cardiogenic shock |
| Patients who have undergone PCI within 30 days prior to the procedure or are scheduled to undergo PCI within 30 days after the procedure |
| Patients with a left ventricular ejection fraction of less than 40% within 30 days prior to the procedure |
| Patients with New York Heart Association Class III or IV heart failure |
| Patients who have undergone PCI within 30 days prior to the procedure or are scheduled to undergo PCI within 30 days after the procedure |
| Patients with evidence of acute myocardial infarction (ST-elevation myocardial infarction [STEMI] or non-STEMI) within 30 days prior to the procedure (creatine phosphokinase [CK] level greater than twice the institutional reference value or CK-MB elevation above the institutional upper limit) |
| Patients who have experienced a stroke or transient ischemic attack within six months prior to the procedure |
| Patients not eligible for emergency coronary artery bypass grafting (CABG) |
| Patients with known contraindications or hypersensitivity reactions to aspirin, heparin, clopidogrel, ticlopidine, prasugrel, or ticagrelor |
| Patients with a platelet count less than 100,000/μL |
| Patients with a history or complication of peptic ulcer or gastrointestinal bleeding within six months prior to the procedure |
| Patients with concomitant diabetes that is poorly controlled with medication |
| Patients allergic to contrast agents who cannot be adequately managed with premedication |
| Patients who are pregnant or breastfeeding at the time of consent |
| Patients with a concomitant medical condition with a life expectancy of less than 12 months |
| Patients participating in another clinical trial at the time of consent |
| Patients with a lesion (stenosis >50%) in the unprotected left main coronary artery |
| Patients whose target lesion is in a vessel that can only be reached through the saphenous vein or arterial bypass graft (including in arterial bypass grafts) |
| Patients with a target lesion located in or distal to a vessel bend greater than 45 degrees |
| Patients with excessive tortuosity in the target vessel |
| Patients with thrombus or ulceration (by angiography or intravascular imaging) in the target vessel |
| Patients with angiographic evidence of severe dissection (type D or greater dissection in the National Heart, Lung and Blood Institute classification) in the target vessel after conventional balloon dilation |
| Patients deemed inappropriate to participate in the trial by the attending physician |

## Table 2. Angiographic analysis core lab evaluation items

All core lab evaluation items are as follows.

- Target lesion (coronary artery segment by the American Heart Association [AHA])
- Lesion type (at the start of the procedure)

AHA lesion classification, in-stent restenosis lesion, ostial lesion, bifurcation lesion, concentricity, bend, calcification, tortuosity, thrombus, dissection, aneurysm

- Presence and degree of dissociation (after KCB01 dilation)
- In-segment minimum lumen diameter
- In-segment residual restenosis

## Table 3. Definitions

Major cardiac adverse events were defined as a composite of cardiac death, myocardial infarction, and target vessel revascularization (TVR).

Cardiac death was defined as any death directly attributable to the heart (myocardial infarction, low cardiac output, heart failure, fatal arrhythmia, etc.), death without witnesses, death of unknown cause, or all deaths of procedural origin, including related treatments.

TVR was defined as a narrowing of the lumen of the target vessel by at least 50% (as assessed by quantitative coronary angiography), observed recurrent anginal symptoms presumed to be related to the target vessel, observed signs of ischemia at rest (electrocardiogram [ECG] changes) or equivalent signs during exercise stress presumed to be related to the target vessel, invasive functional abnormal values are observed on invasive functional tests (e.g., doppler flow velocity reserve or fractional flow reserve), or target lesion stenosis of 70% or greater (as assessed by coronary angiography) in the absence of recurrent ischemic symptoms or ECG changes. PCI or CABG of the target vessel was performed in the absence of recurrent ischemic symptoms or electrocardiographic changes.

Post-procedure myocardial infarction was defined as CK-MB > 5 times the upper limit of the institution’s reference value, observation of symptoms suggestive of myocardial ischemia (such as ischemic chest pain lasting > 20 min), new ischemic changes (ST segment or new abnormal Q waves), new left bundle branch block on electrocardiography, and angiographic evaluation of procedural complications. Angiographic findings were consistent with procedural complications (loss of patency of a major coronary artery or side branch, persistent slow flow, no reflow, or embolism), new loss of viable myocardium, or new localized wall motion abnormalities on imaging studies.

## Table 4 Lesion characteristics of the procedural failure cases

Supplementary table

| Lesion characteristics of the procedural failure cases　　　　　　　　　　　　　　　　　　n=9 | |
| --- | --- |
| Ostial lesion | 1 |
| Bifurcation lesion | 4 |
| Calcification |  |
| None/mild | 0 |
| Moderate | 2 |
| Severe | 7 |
| Bend of lesion |  |
| <45° | 9 |
| 45°~90° | 0 |
| 90°≦ | 0 |
| Tortuosity |  |
| None | 9 |
| Moderate | 0 |
| Severe | 0 |
| Eccentric lesion | 1 |
| ISR | 2 |
| AHA lesion classification |  |
| A | 0 |
| B1 | 1 |
| B2 | 6 |
| C | 2 |

## Table 5. List of all adverse events

| Adverse event | Severity | Seriousness | Causal relationship with KCB01 | Reason for “no relationship” |
| --- | --- | --- | --- | --- |
| Rash | Non-severe | Moderate | No | Due to contrast medium |
| Headache | Non-severe | Mild | No | Due to the treatment position |
| Myocardial necrosis marker increased | Non-severe | Mild | No | Because of possible contrast delay in the diagonal branch due to stenting |
| Device breakage | Non-severe | Mild | No | Caused by the orbital atherectomy system used for treating non-target lesions |
| Coronary artery dissection | Non-severe | Mild | No | Due to rupture of the other balloon |
| Chest discomfort | Non-severe | Mild | No | Transient related to adverse events during treatment of non-target lesions |
| Blood creatine phosphokinase MB increased | Non-severe | Mild | Probably no | Possibly due to concomitant debulking device (Orbital atherectomy system) |
| Bradycardia | Non-severe | Mild | No | Because it was not during or immediately after KCB01 dilation |
| Puncture site hemorrhage | Non-severe | Mild | No | Previous catheterization produced the same event |
| Coronary artery perforation | Non-severe | Mild | No | Due to the post-dilation procedure |
| Erythema | Non-severe | Mild | No | Determined that it was a side effect of the contrast medium |
| Presyncope | Non-severe | Mild | No | Determined that stress due to being nervous was the cause |
| Chest pain | Non-severe | Mild | No | Occurred at time other than KCB01 insertion |

## Table 5 Lesion characteristics of the procedural failure cases

Supplementary table

| Lesion characteristics of the procedural failure cases　　　　　　　　　　　　　　　　　　n=9 | |
| --- | --- |
| Ostial lesion | 1 |
| Bifurcation lesion | 4 |
| Calcification |  |
| None/mild | 0 |
| Moderate | 2 |
| Severe | 7 |
| Bend of lesion |  |
| <45° | 9 |
| 45°~90° | 0 |
| 90°≦ | 0 |
| Tortuosity |  |
| None | 9 |
| Moderate | 0 |
| Severe | 0 |
| Eccentric lesion | 1 |
| ISR | 2 |
| AHA lesion classification |  |
| A | 0 |
| B1 | 1 |
| B2 | 6 |
| C | 2 |
